# Location of a Dexamethasone Implant at the Macula after Intravitreal Injection in a Silicone Oil-Filled Eye

**DOI:** 10.1155/2016/5107652

**Published:** 2016-11-23

**Authors:** Cenap Mahmut Esenulku, Murat Gunay

**Affiliations:** ^1^Department of Ophthalmology, Trabzon Kanuni Training and Research Hospital, Trabzon, Turkey; ^2^Department of Ophthalmology, Trabzon Fatih State Hospital, Trabzon, Turkey

## Abstract

Here, we report a case with cystoid macular edema (CME) due to central retinal vein occlusion (CRVO) presented with a dexamethasone implant (Ozurdex) trapped at the macula in her silicone oil- (SO-) filled eye after injection. No additional complications such as intraocular pressure (IOP) rise or retinal damage were observed. The CME was resolved during the follow-up period. At the last visit, 3 months following the injection, Ozurdex implant was found to be mostly dissolved without any additional ocular complications.

## 1. Introduction

Macular edema (ME) is the most common cause for decreased visual performance in patients with retinal vein occlusion (RVO). Elevated levels of vascular endothelial growth factor (VEGF) and breakdown of the blood-retinal barrier with subsequent release of inflammatory mediators contribute to the development of ME in RVO [1]. Apart from the intravitreal anti-VEGF therapy, Ozurdex (dexamethasone 0.7 mg) has been approved in the form of an intravitreal implant for the treatment of ME associated with RVOs [2].

Afshar et al. [3] firstly described a case with Ozurdex implant on the macula after surgical repair for recurrent retinal detachment with silicone oil (SO) administration. In that report, the implant remained trapped between silicone oil and retinal surface and then moved away from the macula remaining as a pigmented epiretinal membrane. Studies then showed several abnormalities related to intravitreal dexamethasone implant in SO-filled eyes [4–6]. In the present report, we aimed to demonstrate the clinical course of a case with ME due to central RVO (CRVO) presenting with Ozurdex implant on the macula in a SO-filled eye.

## 2. Case Report

A 55-year-old woman with a history of CRVO presented with reduced visual acuity (VA) in her left eye that persists for one month. In the first visit, her best corrected visual acuity (BCVA) was 20/20 in the right eye and 10/200 in the left eye. She has no systemic abnormality except for hypertension. No abnormality in the anterior segment examination of both eyes was observed. The intraocular pressure (IOP) measurements were 13 mmHg in both eyes. Fundus examination revealed CRVO related findings. On spectral domain optical coherence tomography (SD-OCT, Spectralis HRA+OCT, Heidelberg Engineering, Heidelberg, Germany) analysis, cystoid ME (CME) was detected in the left eye. Fundus Fluorescein Angiography (FFA) examination was compatible with CRVO. The patient had already received 7 doses of intravitreal ranibizumab, 3 doses of intravitreal aflibercept, and an intravitreal dexamethasone implant (Ozurdex, Allergan Inc., Irvine, CA) at our center.

**Figure 1:**
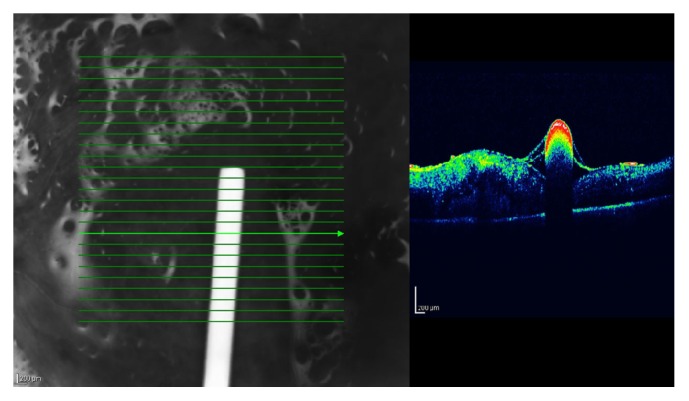
Optical coherence tomography analysis shows hyperreflectance of the intravitreal dexamethasone implant at the fovea with optical shadowing.

After eleven months of the first visit, the patient had undergone a combined procedure of phacoemulsification and pars plana vitrectomy (PPV) with SO injection at another center for persisting CME. Patient was admitted to our hospital with visual complaints in the left eye one week after the surgery. The BCVAs were 20/20 and 5/200 in the right and left eyes, respectively. CME was still present on OCT examination. Intravitreal Ozurdex injection was considered. On postinjection first day, the patient complains about a central linear scotoma in her left eye. On fundus examination, the implant was detected at the macula on the fovea (Figure 1). Clinical observation was considered. The implant gradually dissolved during 3 months. No recurrence in CME was detected during the follow-up. No additional complication was noted. The intravitreal implant was found to be mostly dissolved 3 months following the injection and no CME was detected. Central linear scotoma was relieved after dissolution of the implant.

## 3. Discussion

It was stated that intravitreal injection of an implant may not be suitable in a SO-filled eye. Several concerns on this issue have been proposed including a possible increase in IOP due to volume expansion, improper dissolution of the drug in SO, and prevention of the diffusion of the drug to macula [7]. Kim et al. [5] implanted Ozurdex intravitreally after vitrectomy for a chronic uveitis case in a SO-filled eye. They suggested the use of intravitreal dexamethasone implant in SO-filled vitrectomized eyes in refractory ME secondary to chronic uveitis. We observed successful regression of CME after Ozurdex in the present case. Regression of CME in a SO-filled eye in our case has shown a benefit for patients treated with intravitreal Ozurdex.

Wai Ch'Ng et al. [8] reported the displacement of an intravitreal Ozurdex implant to the anterior vitreus cavity 2 weeks after the implantation. The authors attributed this complication to the slow migration of the implant to the hyaloid fossa due to the absence of posterior vitreous detachment in the patient. Banerjee et al. [6] reported a similar case in a pseudophakic SO-filled eye in which the implant was trapped between the posterior capsule and anterior of the SO bubble. They suggested the SO's buoyancy force as a contributing factor to the implant position in a vitrectomized SO-filled eye. In both cases, no further adverse events were noted. In our case, the implant was detected on the macula one day after the injection similar to the case reported by Afshar et al. [3]. However, we did not observe an ERM formation at location of the implant as seen in that study. The possible explanation for the trapping of an intravitreal implant at the macula in a SO-filled eye was suggested to be the static environment between the SO and fluid space on the retinal surface; thus, the implant may be immobilized at the same position during a period of time [3]. This was 1 month for the previous case and 3 months for our case.

In an experimental study, it has been shown that velocity of the dexamethasone implant is slower in vitreous than in water and that decreases exponentially over distance in vitreous [9]. Differences in velocity might be related to the viscosity of media filled in the eye. The velocity of the dexamethasone implant might be lower in SO-filled eye due to high viscosity of SO.

In conclusion, the presence of the intravitreal dexamethasone implant at the macula in a SO-filled eye after the injection might be a common complication. Physicians should keep in mind this possible phenomenon while administering intravitreal dexamethasone implant in a SO-filled eye. In our opinion, further studies are needed in order to better understand the underlying mechanism of this kind of complication.
